# Implementing a successful journal club in an anesthesiology residency program

**DOI:** 10.12688/f1000research.2-15.v1

**Published:** 2013-01-16

**Authors:** Nathaniel D Pitner, Chris A Fox, Matthias L Riess

**Affiliations:** 1 Department of Anesthesiology, Medical College of Wisconsin, Milwaukee, WI, USA; 2 Department of Physiology, Medical College of Wisconsin, Milwaukee, WI, USA; 3 Clement J Zablocki, V A Medical Center, Milwaukee, WI, USA

## Abstract

Journal clubs are an integral element of residency training. We report the successful implementation of a monthly structured journal club in our anesthesia residency program. Based on resident surveys before and one year after its start, the journal club led to a significantly higher confidence in how to critically appraise literature and present a manuscript. The journal club also improved the residents' ability to search the literature and their statistical knowledge, skills that are essential in the practice of evidence-based medicine. We describe key features that may aid other training programs in organizing a stimulating an educational and sustainable journal club.

## Introduction

Journal clubs are an integral element of residency training across many medical specialties ^1– 5^. Ideally, they teach research design, statistical methods, and critical appraisal skills. Further goals are keeping up-to-date with the current literature and impacting clinical practice through evidence-based medicine ^4^. However, there appears to be no gold standard and organizing a successful journal club that meets these goals and achieves both longevity and high resident participation can be challenging ^3, 4^. Thus, we surveyed the residents in our program to facilitate the creation of a successful journal club and to assess its effectiveness in teaching research methodology and critical appraisal skills.

## Methods

After approval by the Institutional Review Board (PRO9864), a voluntary and anonymous resident survey was conducted initially, and repeated one year after implementation of a structured monthly journal club. Thirty-five (67%) of 52 available residents attended the first journal club meeting. Thirty residents (86%) participated in the initial survey, and 21 residents (95% of the 22 attending) completed the follow-up survey one year later [follow-up data in brackets].

Results are expressed as mean ± SEM (standard error of the mean) or percentages. Residents were asked to judge the usefulness of the journal club for their training on a scale of 1 (not useful) to 5 (extremely useful) as well as their abilities in four separate areas (literature search, critical literature appraisal, manuscript presentation, and statistics) on a scale of 1 (not comfortable) to 5 (extremely comfortable). Kruskal-Wallis and Mann-Whitney tests were utilized for statistical analysis of these rating-scale data. Chi square test was used to analyze categorical data, e.g. preference of mandatory vs. voluntary attendance, frequency, time and location of journal club meetings, and preferred type of articles. Spearman’s rank correlation was employed to determine the correlation between two variables. Bonferroni correction was applied to correct for multiple comparisons. Statistical significance was assumed when P < 0.05 (two-tailed). Significance symbols are * vs. alterative, † vs. initial survey.

## Results

Having a regular and structured journal club was continuously rated to be useful at 3.5±0.2 [3.3±0.2]. In total, 70%* [62%] of residents favored monthly, 17% [38%] quarterly, and 13% [0%] weekly journal club meetings. A total of 63%* [43%] preferred voluntary, and 37% [57%] mandatory attendance; level of training correlated positively with a shift to mandatory while perceived usefulness did not. A total of 53% [38%] of residents preferred to meet before work, 40% [29%] after work; 57% [57%] preferred the workplace, 40% [33%] outside the workplace; time (before work) correlated significantly with location (workplace). In total, 30% [24%] preferred the selection of review articles, 23% [5%] primary research studies, and 43% [71%] a mix of both types for discussion; interest in primary studies correlated positively with level of training.

**Figure 1.**
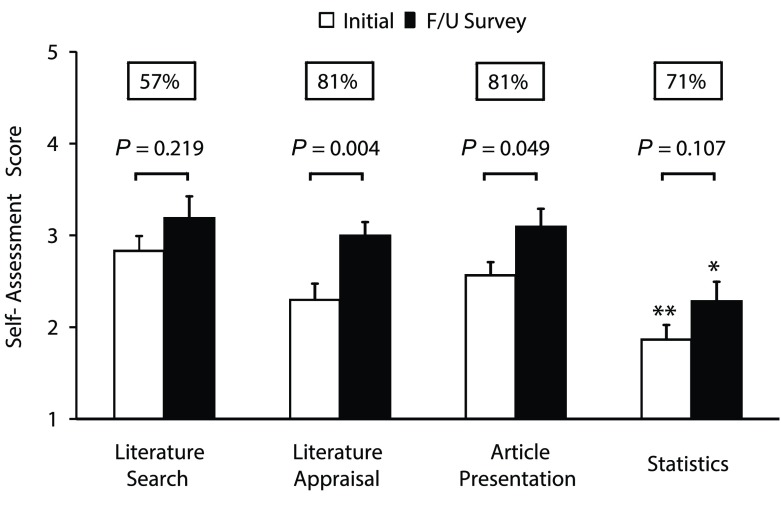
Shows the residents’ ratings of their ability to search literature, to critically appraise literature, to present a manuscript, and their statistical knowledge before (white bars) and one year after implementation of a structured monthly journal club (black bars). Self-assessment scores range from 1 (not comfortable) to 5 (extremely comfortable). Data are presented as mean ± SEM. Percentages state the fraction of residents who reported in the follow-up survey to have profited from journal club in these four areas. *P* values are given for comparisons between initial and follow-up surveys. Statistical significance was assumed when *P* < 0.05 (two tailed). Significance symbols are * vs. literature search, ** vs. literature search and manuscript presentation.

67% of responders in the initial survey reported their expectations: 85% preferred articles that had an impact on their clinical decision-making, while 50% wanted to improve their skills in critical appraisal, 20% literature search, 15% manuscript presentation, and 15% statistical methods. Between 1 (not comfortable) and 5 (extremely comfortable), residents rated their ability to search literature 2.8±0.2 [3.2±0.2], to critically appraise lit erature 2.3±0.2 [3.0±0.1†], to present a manuscript 2.6±0.1 [3.1±0.2†], and their statistical knowledge 1.9±0.2* (vs. search and presentation) [2.3±0.2* (vs. search)] (Figure 1). In the follow-up survey, the majority of residents reported a benefit from the journal club in each of these four areas (Figure 1). Interestingly, perceived statistical knowledge correlated significantly with length of previous statistical training, but in-training exam results in statistics did not.

33% in the follow-up survey favored a single faculty moderator for the entire year, having a different moderator for each meeting was suggested by 52% and having multiple moderators per meeting by 14%* (vs. different moderators).

Journal Club Survey The raw data of the residents survey from the structured monthly journal club.

## Discussion

Journal clubs have long been recognized as an effective tool for teaching evidence-based medicine ^2^. They are often used to teach clinical epidemiology and biostatistics to foster critical appraisal skills, an educational goal that is frequently rated higher than keeping up with the medical literature ^3, 5, 6^. Residents who are being taught appraisal skills reportedly pay more attention to a study’s methodology and are more critical of its conclusions ^3^.

Factors associated with high attendance and longevity includes mandatory attendance, incentives such as food, and a high educational value attributed by the program director. A trained journal club leader to choose articles and direct the discussion leads to higher educational satisfaction ^3, 4^. Interestingly, presentation of original articles only is associated with longevity, but lower attendance rates ^7^. Other important characteristics of successful journal clubs are timely dissemination of reading material, preferably via the internet, and regularly scheduled meetings at predictable intervals and at a time appropriate for all participants ^4^; in this context, monthly intervals have been found optimal because of a potentially diminishing interest if conducted too often.

Following these recommendations we have successfully implemented a structured Faculty-led journal club with presentation and critical evaluation by a group of two to three second-year residents. In both surveys this was well received and considered very useful. The journal club is held monthly at the workplace and before work, and attendance is mandatory; free food is available. Based on follow-up data, we have switched from initially one faculty moderator for the entire year to now having a different moderator from a variety of anesthesia subspecialties for each meeting. A mix of reviews and seminal original articles is selected by the moderators, and particular attention to methodology and statistics is emphasized. The journal club led to significantly higher ratings in the residents’ ability to critically appraise literature and to present a manuscript, and improvement in literature search skills and statistical knowledge.

Although we have to acknowledge the natural limitations of our single-center study – such as a limited sample size and further to be determined discriminative validity, and inter- and intra-rater reliability – its results show that a journal club can be an excellent teaching tool that complements other theoretical and practical training in anesthesiology, and provides a skill set necessary to understand and practice evidence-based medicine. Our experience may aid and encourage other training programs in organizing a stimulating, educational and sustainable journal club that is well-received by its residents and can accomplish these goals.

## Consent

This case report is in accordance with Institutional Review Board guidelines and IRB approval was obtained before beginning of the study (PRO9864).
