# Supplementary material for: Implementing a successful journal club in an anesthesiology residency program
Source: F1000Res. 2013 Jan 16;2:15. [Version 1] doi: 10.12688/f1000research.2-15.v1 (PMC3752701; doi:10.12688/f1000research.2-15.v1)
Supplement: Peer review history

# Peer review: Referee response for version 1

**Published:** 2013-01-30

**Authors:** Patricia Khashayar

**Affiliations:** Research and Development Center, Sina Hospital, Tehran, Iran

I have read this submission. I believe that I have an appropriate level of expertise to confirm that it is of an acceptable scientific standard.

# Peer review: Referee response for version 1

**Published:** 2013-01-29

**Authors:** Andrew Lee

**Affiliations:** Department of Ophthalmology, University of Iowa Hospitals and Clinics, Iowa City, IA, USA

I have read this submission. I believe that I have an appropriate level of expertise to confirm that it is of an acceptable scientific standard.

# Peer review: Referee response for version 1

**Published:** 2013-01-18

**Authors:** Bob Phillips

**Affiliations:** Centre for Reviews and Dissemination, University of York, York, UK

I have read this submission. I believe that I have an appropriate level of expertise to confirm that it is of an acceptable scientific standard.
